# From Phenotype to Genotype and Beyond: Insights into Familial Hypercholesterolemia and Familial Hypertriglyceridemia

**DOI:** 10.3390/medicina62071257

**Published:** 2026-06-29

**Authors:** Dragos Cozma, Daniel Florin Lighezan, Cristina Tudoran, Oana Raluca Voinescu, Cristian Mornos

**Affiliations:** 1 Institute of Cardiovascular Diseases Timisoara, 300310 Timisoara, Romania; dragos.cozma@umft.ro (D.C.); mornos.cristian@umft.ro (C.M.); 2 Cardiology Department, “Victor Babeș” University of Medicine and Pharmacy, 300041 Timisoara, Romania; 3 Research Center, Institute of Cardiovascular Diseases Timisoara, 300310 Timisoara, Romania; voinescu.oana@umft.ro; 4 Emergency City Hospital Timisoara, Gheorghe Dima Street Nr. 5, 300254 Timisoara, Romania; dlighezan@umft.ro; 5 Centre of Advanced Research in Cardiology and Hemostasology, Faculty of the “Victor Babeș” University of Medicine and Pharmacy, E. Murgu Square, Nr. 2, 300041 Timisoara, Romania; 6 Department V, Internal Medicine I—Discipline of Medical Semiology I, “Victor Babeș” University of Medicine and Pharmacy, E. Murgu Square, Nr. 2, 300041 Timisoara, Romania; 7 Department VII, Internal Medicine II, Discipline of Cardiology, “Victor Babeș” University of Medicine and Pharmacy, E. Murgu Square, Nr. 2, 300041 Timisoara, Romania; 8 County Emergency Hospital “Pius Brinzeu”, L. Rebreanu, Nr. 156, 300723 Timisoara, Romania; 9 Center of Molecular Research in Nephrology and Vascular Disease, “Victor Babeș” University of Medicine and Pharmacy, E. Murgu Square, Nr. 2, 300041 Timisoara, Romania; 10 Department VI—Cardiology, “Victor Babeș” University of Medicine and Pharmacy, E. Murgu Square, Nr. 2, 300041 Timisoara, Romania

**Keywords:** familial hypercholesterolemia, familial hypertriglyceridemia, LDL cholesterol, tryglicerides

## Abstract

Familial hypercholesterolemia (FH) and familial hypertriglyceridemia (FHTG) represent a spectrum of inherited conditions with profoundly different etiologies, risk profiles, and therapeutic implications. Despite decades of clinical experience, their formal diagnostic definitions remain rooted in frameworks developed before the genomic era (the Dutch Lipid Clinic Network (DLCN) score), leading to substantial gaps in diagnostic accuracy. This review traces the historical evolution of diagnostic criteria for FH and FHTG from early phenotypic observation to contemporary genomic and biomarker-driven models. It systematically evaluates the major limitations of current criteria, including the (DLCN) score, and integrates evidence from landmark Mendelian randomization (MR) studies to identify persistent gaps. A narrative synthesis of landmark clinical, epidemiological, and genetic studies was performed, encompassing the original discovery of the low-density lipoprotein cholesterol (LDL-C) receptor pathway, the development of international diagnostic criteria, and contemporary mendelian randomization (MR) evidence on the causal roles of LDL-C, lipoprotein (a) [Lp(a)], triglyceride-rich lipoprotein remnants, and apolipoprotein B (ApoB). Current diagnostic frameworks suffer from age-dependent confounding of LDL-C measurements, failure to account for Lp(a)-mediated phenocopies, inadequate discrimination between monogenic and polygenic etiologies, sex differences, ethnicity, and inapplicability to pediatric populations. MR data reveal that the causal architecture of cardiovascular risk in these disorders is particle-centric (ApoB) rather than LDL-C-centric, and that remnant cholesterol, not triglyceride per se, drives atherosclerotic cardiovascular disease risk in FHTG. We evidenced the evolution of treatment options and the morbidity and mortality rates for FH and FHTG from the 1970s until the 2020s. Future diagnostic paradigms should integrate lifetime Lp(a) measurement, polygenic risk scoring, ApoB quantification, and cascade genomic testing to replace phenotype-only approaches. This review concludes by proposing a four-step integrated diagnostic algorithm for FH and FHTG.

## 1. Introduction

**Figure 1:**
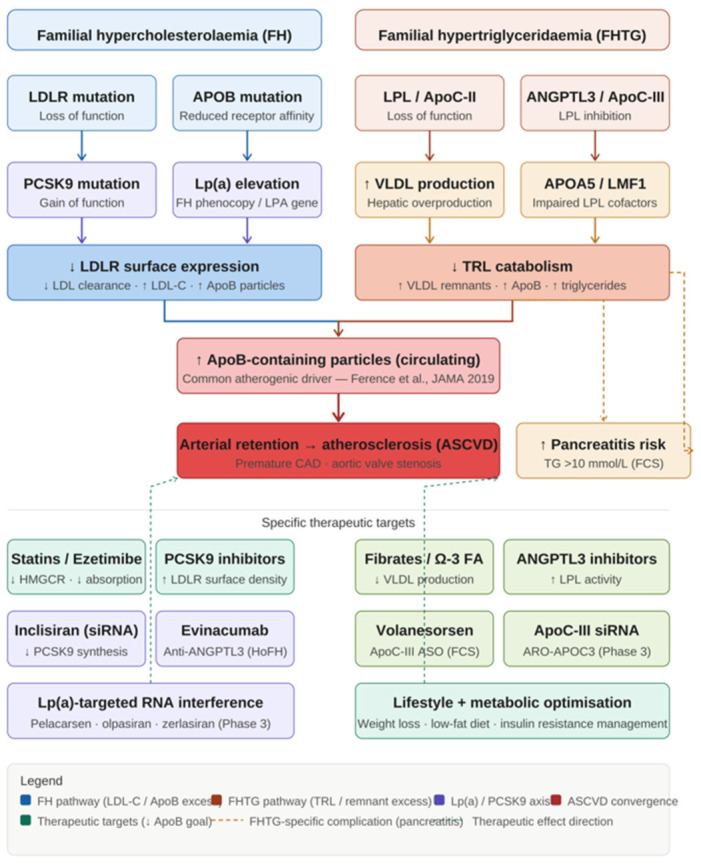
Underlying pathophysiology and specific therapeutic targets [8]. Legend: ANGTL3—angiopoietin-like protein 3; APOB—the gene encoding apolipoprotein B; APOA5—gene coding apolipoprotein A5; Apo B—apolipoprotein B; Apo C III—apolipoprotein C III; ARO-APOC3—an investigational RNA-interference therapeutic designed to lower triglycerides by silencing the APOC3 gene; ASO—antisense oligonucleotide; CAD—coronary artery disease; FCS—familial chylomicronemia syndrome; HMGCR—3-hydroxy-3-methylglutaryl-coenzyme A reductase;; LDL—low-density lipoprotein; LDLR—low-density lipoprotein receptor; Lp(a)—lipoprotein a; LPL—lipoprotein lipase; RNA—ribonucleic acid; PCSK9—proprotein convertase subtilisin/kexin type 9; TG—triglyceride; TRL—triglyceride-rich lipoprotein; VLDL—very low-density lipoprotein. This figure was designed with BioRender (https://www.biorender.com/).

The term “hereditary hyperlipidemia” is a broad clinical and genetic category containing inherited disorders characterized by abnormal plasma lipid concentrations, and it encompasses several metabolic disorders: monogenic familial hypercholesterolemia (FH), polygenic hypercholesterolemia, familial combined hyperlipidemia, familial dysbetalipoproteinemia, sitosterolemia, monogenic familial chylomicronemia syndrome (FCS), and polygenic familial hypertriglyceridemia (FHTG). FH and FHTG are the most studied of these entities because, together, they represent the major inherited disorders affecting the two principal lipid fractions associated with very high cardiovascular disease risk: low-density lipoprotein cholesterol (LDL-C) and triglycerides. FH, characterized by lifelong elevation of LDL-C, determines markedly accelerated atherosclerosis, greatly increasing the risk of premature atherosclerotic cardiovascular disease (ASCVD) and cardiovascular mortality. FHTG, characterized by a persistent elevation in circulating triglycerides, is connected to metabolic dysfunction, insulin resistance, and increased cardiovascular risk in many patients. FH and FHTG have the highest incidence and the greatest risk of premature ASCVD from all pathologies included in “hereditary hyperlipidemia” [1,2]. The pathophysiological mechanisms of FH and FHTG are complex and sometimes intertwined (see Figure 1).

FH and familial hypertriglyceridemia FHTG are among the most prevalent inherited disorders in human medicine, yet both remain substantially underdiagnosed in clinical practice. FH, caused predominantly by loss-of-function mutations in the LDL receptor (LDLR) gene, is estimated to affect approximately 1 in 250 individuals worldwide, a global burden of approximately 32 million people, yet fewer than 10% carry a formal diagnosis in most countries [3,4]. FHTG, a heterogeneous condition driven by polygenic susceptibility interacting with metabolic stressors, is similarly under-recognized, particularly when triglyceride elevations remain below the threshold for acute pancreatitis [5,6].

The diagnostic challenge in both conditions is compounded by the historical reliance on phenotypic surrogates—elevated LDL-C, physical stigmata such as tendon xanthomas, and premature cardiovascular events—as proxies for a fundamentally genetic diagnosis. These surrogates were adequate when genetic testing was unavailable. Still, they introduce systematic biases that worsen with advancing patient age, early statin use, and the growing prevalence of metabolic syndrome, each of which distorts the lipid phenotype in ways that mimic or obscure the primary genetic defect [6,7].

Mendelian randomization (MR) studies, leveraging the natural randomization of genetic variants across populations, have simultaneously clarified the causal roles of specific lipoprotein fractions and exposed the limitations of cholesterol-centric rather than particle-centric risk assessment [8,9]. These developments, combined with the imminent arrival of ribonucleic acid (RNA) interference therapies targeting lipoprotein (a) (Lp(a)) and the broadening availability of polygenic risk scoring, are converging toward a new diagnostic paradigm that has yet to be formally incorporated into international guidelines [4,7,10].

This review synthesizes the historical trajectory of diagnostic criteria for FH and FHTG, evaluates the evidentiary gaps that define current practice, and proposes a forward-looking, integrated model to modernize the current definitions of FH and FHTG. The structure proceeds chronologically, from phenotypic observation through genetic discovery to the present genomic era, before addressing the specific shortcomings of contemporary tools and the evidence that these methods should be improved.

## 2. A Historical Synopsis on the Diagnosis of FH and FHTG

### 2.1. Phase I: The Phenotypic Era (Pre-1970s)—When the Body Was the Diagnostic Tool

Before the molecular biology revolution, the identification of heritable hyperlipidemias was necessarily clinical. Clinicians in the early twentieth century observed that certain patients exhibited a constellation of findings—dramatically elevated cholesterol levels, subcutaneous lipid deposits (xanthomas), and coronary artery disease presenting decades earlier than expected—that appeared to cluster in families with a pattern consistent with autosomal-dominant inheritance [11].

The classic triad of FH—markedly elevated LDL-C, tendon xanthomas, and premature coronary heart disease—formed the empirical bedrock of diagnosis. This triad reflected the end of the phenotypic spectrum, effectively limiting clinical recognition to the most severely affected individuals: those with homozygous FH, who expressed all three features before the third decade of life, and severely affected heterozygotes, who might manifest the triad by the fifth or sixth decade [11].

The implications of this observational framework were significant. First, the invisible majority of FH heterozygotes—those with elevated LDL-C but absent xanthomas and no premature cardiovascular event—went unrecognized. Second, the biological distinction between heritable cholesterol excess and diet-induced hypercholesterolemia remained conceptually blurred; both produced elevated cholesterol, and in the absence of a family history of hypercholesterolemia, differentiation was imprecise. Third, because the diagnosis could be confirmed only post hoc—after the cardiovascular event had occurred—the opportunity for preventive measures, that FH uniquely presents, was systematically missed [5,12].

For FHTG, the clinical picture in the pre-molecular era was similarly dominated by its most dramatic manifestation: severe hypertriglyceridemia producing eruptive xanthomas, lipemia retinalis, and acute pancreatitis. This phenotype, later representing the FCS in its monogenic form, was distinguishable from milder polygenic hypertriglyceridemia only by the severity and persistence of hypertriglyceride and its relative refractoriness to dietary modification [2,5].

Before modern treatment, FH was one of the strongest known inherited risk factors for premature coronary artery disease. Untreated men with heterozygous FH had at least a 50% cumulative risk of coronary heart disease by age 60, and untreated women had about a 30% cumulative risk by age 60. The mortality rates were particularly striking in young adults [13,14]. Coronary mortality was nearly 100-fold higher than in the general population among FH patients aged 20–39 years. In those aged 40–59 years, coronary mortality was approximately 4-fold higher than expected. Many patients experienced myocardial infarction in their 30s–50s [14].

Homozygous FH had a far worse prognosis. Severe aortic and coronary atherosclerosis often developed during childhood [15]. Myocardial infarction could occur in the teenage years. Many patients died before age 30, and some before age 20, from cardiovascular disease. In practical terms, homozygous FH was often a fatal childhood or young-adult disease before the 1970s [16].

For most individuals with FHTG, the overall life expectancy was often near normal if pancreatitis did not occur [2]. Mortality was driven primarily by severe or recurrent pancreatitis rather than coronary disease [17]. Before modern intensive care, severe pancreatitis carried substantial mortality (often reported in historical series as 10–30% or higher for severe necrotizing disease), but this reflected the complication rather than the genetic disorder itself [18].

Therapeutic options were limited in this period. FH was treated with dietary therapy (restriction of saturated fats and cholesterol, increased intake of polyunsaturated fats, and weight control), but with limited effect on LDL-C levels. Medical therapy consisted of bile acid sequestrants (Cholestyramine and Colestipol) and nicotinic acid (niacin) [19]. In specialized centers, in severe cases, partial ileal bypass and lipoprotein apheresis (LDL apheresis) were performed.

FHTG was treated by dietary regimes: restriction of simple sugars and refined carbohydrates, cessation of alcohol consumption, weight reduction, and, in severe cases, low-fat diets. Medical therapies included clofibrate and nicotinic acid [20].

### 2.2. Phase II: The Genetic Breakthrough (1970s–1980s)—The LDL Receptor and the Mechanistic Definition

The foundational transformation in understanding FH came from the experimental work of Joseph Goldstein and Michael Brown at the University of Texas Southwestern Medical Center in the 1970s. Through a series of elegant cell biology experiments in cultured human fibroblasts, they identified, characterized, and delineated the function of the LDL receptor (LDLR)—the cell-surface protein responsible for binding and internalizing LDL particles to regulate intracellular cholesterol homeostasis [21].

Their discovery, through which they received the Nobel Prize in Physiology or Medicine in 1985, fundamentally reframed FH from a high-cholesterol phenotype to a cellular defect in LDL clearance [21]. The pathophysiological sequence became clear: loss-of-function mutations in LDLR result in reduced capacity of hepatocytes and peripheral cells to bind and internalize LDL particles, inducing a compensatory upregulation of endogenous cholesterol synthesis and a sustained, lifelong elevation of circulating LDL cholesterol (LDL-C) from a very young age. In heterozygotes, residual receptor activity of approximately 50% induces moderate LDL elevation (typically 5–10 mmol/L in untreated adults); in homozygotes, with near-absent receptor function, LDL-C routinely exceeds 13 mmol/L [21,22].

Critically, the genetic definition established by Goldstein and Brown [21] was mechanistic: FH was a disease of defective LDL receptor function, not simply of elevated cholesterol. Subsequent decades revealed that the same phenotype could be produced by gain-of-function mutations in proprotein convertase subtilisin/kexin type 9 (PCSK9)—a serine protease that promotes LDLR degradation—and by mutations in apolipoprotein B (ApoB) genes (APOB), which reduce LDL particle affinity for its receptor [23,24]. The existence of several pathophysiological pathways became very important for the diagnosis of FH, as it determines prognosis and treatment response while being phenotypically indistinguishable.

For hypertriglyceridemia, another genetic discovery of the 1970s–1980s identified deficiencies in lipoprotein lipase (LPL) and its cofactor apolipoprotein C-II (ApoC-II) as causes of severe monogenic hypertriglyceridemia, establishing the enzymatic framework for chylomicronemia [5,7]. The recognition that triglyceride-rich lipoprotein metabolism was distinct from LDL metabolism—involving different enzymes, receptors, and metabolic pathways—began to differentiate FH from FHTG as separate biological entities rather than variants of the same problem [25].

The mortality through coronary artery disease was 3.9-fold higher in patients with heterozygous FH compared to the general population [26]. In patients with FH, all-cause mortality was about 1.8-fold higher than in the general population.

By age 60, the cumulative risk of a coronary event was estimated at over 50% in men and around 30% in women with heterozygous FH [14]. Premature myocardial infarction, angina, and need for coronary revascularization were common manifestations. During the 1970s–1980s, FH was primarily a disease of premature atherosclerotic cardiovascular disease, with especially severe excess mortality in young adults [23,27].

In this phase, morbidity and mortality data for FHTG are less clear because historical studies often grouped FHTG together with other inherited hyperlipidemias, such as familial combined hyperlipidemia. Patients with FHTG had increased cardiovascular mortality compared to the general population, but the excess risk was considerably smaller and less consistent than that seen in FH [28].

The major concerns in FHTG were severe hypertriglyceridemia, recurrent acute pancreatitis, and chylomicronemia syndrome in the most severe cases. Classic FHTG was generally not associated with the same dramatic rates of premature coronary disease as FH, particularly when LDL cholesterol was not elevated [29].

The therapy for FH did not change much. To the drugs mentioned in phase I, probucol was added, with a modest reduction in LDL-C. Partial ileal bypass remains the main surgical option, and as experimental therapies, LDL apheresis and occasionally portocaval shunt are performed in very severe cases [30].

The therapies for FHTG remained the same as in phase I.

### 2.3. Phase III: Diagnostic Standardization (1990s)—Development of Clinical Scoring Criteria

#### 2.3.1. The Dutch Lipid Clinic Network (DLCN) Criteria

As genetic testing remained expensive and technically demanding throughout the 1990s, the clinical imperative was to develop a validated, accessible tool allowing patients’ stratification by probability of FH without requiring molecular confirmation. Thus, the Dutch Lipid Clinic Network (DLCN) criteria were developed in the Netherlands and subsequently validated internationally [12,31].

The DLCN assigns numerical points across four domains: family history of premature coronary heart disease or elevated LDL-C in first-degree relatives; personal history of premature atherosclerotic disease; physical examination findings (tendon xanthomas or corneal arcus before age 45); and LDL-C levels, with higher concentrations yielding disproportionately greater scores. A total score exceeding 8 points establishes the diagnosis of FH; scores of 6–8, 3–5, and below 3 are defined as probable, possible, or unlikely FH [12].

The DLCN was a substantial methodological advance. It provided a standardized, reproducible probability framework applicable across clinical settings and was designed to be sensitive for the full phenotypic spectrum—not only the classical triad—by incorporating family history as an independent contributor. Its recognition by the European Atherosclerosis Society (EAS) and integration into international cascade screening programs validated its utility as a diagnostic tool applicable to the general population [3,4].

#### 2.3.2. Parallel Criteria: Simon Broome and MEDPED

Concurrently, the Simon Broome Register Group in the United Kingdom developed a categorical classification system distinguishing Definite from Possible FH based on LDL-C thresholds combined with the presence of tendon xanthomas (for Definite) or family history of premature CHD or hypercholesterolemia (for Possible) [26]. The American Make Early Diagnoses to Prevent Early Deaths (MED-PED) program adopted a complementary approach using age- and family-relationship-specific LDL-C cut-offs, enabling rapid screening without the point-scoring complexity of the DLCN [32,33].

These three systems shared a common limitation: they were designed for adult-specific populations with established phenotypic expression. None was calibrated for pediatric application, cascade screening of young relatives, or the integration of biomarkers beyond LDL-C and LDL-C cut-offs by age/relationship (Table 1).

Compared with the pre-statin era, in patients with FH, coronary mortality fell markedly during the 1990s. The mortality in FH patients aged 20–79 years fell from about 3.4 times that of the general population, before 1992, to about 2.1 times after statins were introduced. Among FH patients without established coronary disease (primary prevention), coronary heart disease mortality was reduced to approximately that of the general population after 1992. Even in the 1990s, FH remained associated with premature myocardial infarction, angina, coronary revascularization procedures, and accelerated atherosclerosis. However, the incidence of new coronary events declined substantially after widespread statin use. In treated FH patients, myocardial infarction risk approached that of age-matched controls [35].

Patients with FHTG had an estimated 1.7-fold higher cardiovascular mortality risk compared to the general population. Elevated baseline triglycerides independently predicted later cardiovascular mortality within FHTG families. The major clinical complication for FHTG remained acute pancreatitis, particularly when triglycerides became very high (>500–1000 mg/dL, often much higher during attacks). Chylomicronemia syndrome could occur in severe cases. Unlike FH, classic FHTG was not generally considered a major cause of premature coronary disease when LDL cholesterol was not elevated [36]. The 1990s are characterized by a major breakthrough in the treatment of FH. Statins (lovastatin, simvastatin, pravastatin, fluvastatin, and atorvastatin—in the late 1990s) were developed and became the first-line treatment to lower LDL-C levels. Significant reductions in LDL-C levels are obtained for the first time. Moderate doses of statins reduced LDL values by approximately 20–40%, and with high-potency statins, a reduction of 50% could be achieved. A reduction of 50 to 70% could be achieved with LDL apheresis [37]. Another revolutionary treatment for that period was liver transplantation, which, in some cases, could be curative, as the transplanted liver expresses normal LDL receptors.

Fibrates (gemfibrozil, fenofibrate, and bezafibrate) became the first-line therapy in FHTG. Statins were also used for the FHTG treatment. Omega-3 fatty acids from fish oil were recognized for their triglyceride-lowering properties [38].

### 2.4. Phase IV: The Molecular Genetics Era (2000s)—Genotype–Phenotype Correlations and the Polygenic Revelation

The widespread application of Sanger sequencing and, subsequently, next-generation sequencing technologies in the 2000s brought a critical revelation: many patients meeting clinical DLCN criteria for Definite FH did not carry a causal mutation in LDLR, APOB, or PCSK9 [12,39]. This discordance between clinical phenotype and molecular genotype introduced the concept of polygenic FH phenocopies—individuals with phenotypically similar LDL-C elevations driven not by a single high-impact variant but by the additive effect of many common low-effect-size variants, each individually below the threshold of clinical significance [22].

Talmud and colleagues demonstrated in 2013 that the cumulative effect of a polygenic score (PGS) comprising as few as six validated LDL-raising single-nucleotide polymorphisms (SNPs) could explain a substantial proportion of clinically diagnosed cases with FH [40]. Individuals with high polygenic scores exhibited similar LDL-C levels to those with monogenic FH but a substantially lower absolute cardiovascular risk; a distinction with direct therapeutic implications that the DLCN score cannot make.

This polygenic revelation had three practical consequences. First, it suggested that the current global burden of diagnosed FH likely contains a heterogeneous mix of true monogenic disease and polygenic phenocopies, with different prognoses. Second, it implied that aggressive statin intensification and PCSK9 inhibitor use—justified for monogenic FH—may be disproportionate for polygenic phenocopies with lower intrinsic risk. Third, it highlighted that the DLCN and its equivalents, being phenotype-based, proved unable to make this clinically critical distinction [2,41].

Concurrently, the genetics of hypertriglyceridemia were substantially clarified. Hegele and colleagues demonstrated in a landmark 2014 review that severe hypertriglyceridemia is predominantly polygenic, with most patients carrying a burden of common triglyceride-raising variants compounded by rare heterozygous mutations in LPL, APOC2, APOA5, lipase maturation factor 1 (LMF1), or glycosylphosphatidylinositol-anchored high-density lipoprotein-binding protein (1GPIHBP1) genes [5]. Monogenic familial chylomicronemia syndrome, caused by homozygous or compound heterozygous loss-of-function mutations in LPL or its cofactors, represents a small fraction of cases with extreme triglyceridemia but is clinically distinct for its complete dietary fat sensitivity, severity, and pancreatitis risk [8,21,22,42,43].

FH remained a major cause of premature cardiovascular disease, but widespread statin use substantially reduced morbidity and mortality compared with the 1970s–1980s. In statin-treated FH patients without prior cardiovascular disease, the absolute risk of a cardiovascular event was approximately 3% per year in men and 1.6% per year in women [44].

FHTG continued to be characterized primarily by pancreatitis risk rather than markedly increased cardiovascular mortality [18].

This phase marked a period of major progress in lipid management. Statins became firmly established as first-line therapy for FH, while combination therapy and improved LDL apheresis expanded treatment options for severe cases. The most commonly used agents included: atorvastatin, simvastatin, pravastatin, rosuvastatin (introduced in 2003), fluvastatin, and lovastatin. A major addition during this decade was ezetimibe. It was approved in the early 2000s. It inhibited intestinal cholesterol absorption and was frequently combined with statins for additional LDL reduction. By the 2000s, LDL apheresis was an established treatment in specialized centers [45].

For FHTG, fibrates, omega-3 fatty acids, and better management of metabolic syndrome became standard. Fibrates (fenofibrate, gemfibrozil, and bezafibrate) were the first-line treatment, reducing triglyceride levels by 30 to 50%. Fish oil was increasingly used [46].

### 2.5. Phase V: The Mendelian Randomization Era (2010s)—Establishing Causal Architecture and Exposing Phenotypic Limitations

#### 2.5.1. LDL-C and Coronary Heart Disease: Lifelong Exposure and Disproportionate Benefit

MR uses genetic variants as instrumental variables to establish causal relationships between exposures and outcomes in observational populations, exploiting the principle that allele assignment at conception is analogous to random treatment allocation in a clinical trial and is therefore unconfounded by postnatal lifestyle or environmental factors [9].

The pivotal application of MR to LDL-C was the 2012 meta-analysis by Ference and colleagues, which pooled genetic data from 312,321 participants across 14 MR studies [8]. The fundamental finding was a 54.5% risk reduction in coronary heart disease per 1 mmol/L lower LDL-C when the genetic exposure was present from birth, approximately 2.5-fold greater than the risk reduction per equivalent LDL lowering observed in 5-year statin trials. This disparity directly demonstrated that the duration of LDL exposure is as consequential as its magnitude, providing the first robust causal evidence for early intervention in FH.

The 2016 HMGCR/PCSK9 comparison by Ference and colleagues added methodological precision [8]. By comparing genetic variants in the statin target gene, 3-hydroxy-3-methylglutaryl-coenzyme A reductase (HMGCR), with variants in PCSK9, a target of a subsequently approved drug class, they demonstrated that the cardiovascular benefit per unit of LDL lowering was pathway-independent. This finding validated PCSK9 inhibition as a therapeutic mechanism equivalent to statins, considering cholesterol-mediated risk reduction per mmol/L, supporting the expansion of the FH treatment arsenal before the completion of outcome trials [47].

#### 2.5.2. ApoB as the Causal Determinant: A Paradigm Shift from Cholesterol to Particles

Perhaps the most important finding for the diagnostics of FH and FHTG in the MR era was the 2019 multi-variable MR (MVMR) analysis by Ference and colleagues, published in JAMA, which simultaneously modeled LDL-C, triglycerides, and ApoB [48]. By partitioning the contributions of these co-linear variables using genetic instruments, the study demonstrated that it is the total plasma concentration of ApoB-containing particles—rather than their cholesterol content—that determines atherosclerotic cardiovascular disease risk. LDL-C and triglycerides lost independent predictive value after adjustment for ApoB [48].

This finding has profound diagnostic implications. Both FH and FHTG increase ApoB-containing particle number through different mechanisms—FH by impaired clearance of LDL particles; FHTG by increased production and impaired catabolism of VLDL and its remnants—but conventional clinical criteria for both conditions are calibrated to cholesterol concentrations rather than particle burden. An individual with discordantly low LDL-C but high ApoB (as occurs in metabolic syndrome with small dense LDL predominance) may be at equivalent or even greater risk than an FH patient with elevated LDL-C but lower particle number [48,49].

#### 2.5.3. Lipoprotein(a): From Risk Marker to Causal Cardiovascular Disease Driver

Lp(a) is a structurally unique lipoprotein in which an LDL-like particle is covalently bonded to apolipoprotein(a), encoded by the LPA gene. Plasma Lp(a) concentrations are approximately 90% genetically determined by the number of kringle IV type 2 repeats in the LPA gene, exhibit minimal response to diet, exercise, or conventional lipid-lowering therapy, and reach stable levels at approximately 5 years of age [7].

MR studies have established Lp(a) as an independent causal driver of coronary heart disease and aortic valve stenosis. Clarke and colleagues demonstrated in 2009 using genetic variants in LPA that elevated Lp(a) is causally associated with coronary artery disease risk in a manner not explained by its LDL component [50]. Burgess and colleagues estimated in 2018 that, to achieve equivalent coronary risk reduction to a 1 mmol/L decrease in LDL-C, Lp(a) would need to be reduced by approximately 100 mg/dL, a target only recently approached by RNA-interference agents [51].

The diagnostic relevance of Lp(a) for FH classification is twofold. First, because the standard LDL-C assay measures the cholesterol content of all ApoB-containing particles, a substantial Lp(a) elevation falsely inflates the apparent LDL-C, a phenomenon estimated to affect up to 25% of patients who meet DLCN criteria for Definite or Probable FH [6]. These individuals may not carry LDLR, APOB, or PCSK9 mutations but rather have Lp(a)-mediated phenocopies requiring entirely different management. Second, the co-occurrence of FH and elevated Lp(a) constitutes a double reason to consider the patient at extreme cardiovascular risk, requiring aggressive, early intervention [7].

#### 2.5.4. Triglyceride-Rich Lipoproteins: Remnant Cholesterol as the True Atherogenic Fraction

For hypertriglyceridemia, the MR literature has provided an important conceptual clarification. Varbo et al. demonstrated in 2013 that it is not the triglyceride fatty acid content per se that drives ischemic heart disease but rather the cholesterol carried in triglyceride-rich lipoprotein remnants, the so-called remnant cholesterol. A 1 mmol/L genetic increase in remnant cholesterol was associated with a 2.8-fold increase in coronary artery disease risk, comparable in magnitude to the LDL-C–coronary heart disease relationship [52]. Other studies similarly confirmed, using rare variants in triglyceride-related genes, that hypertriglyceridemia is causally linked to coronary disease [53].

This distinction matters for FHTG diagnosis and management. Measuring triglycerides alone captures the fatty acid burden but not the atherogenic particle burden. Non-HDL cholesterol—which encompasses all ApoB-containing lipoprotein cholesterol, including remnants—and direct ApoB measurements capture more accurately the cardiovascular risk attributable to FHTG than triglyceride concentrations per se [8,11,22].

#### 2.5.5. HDL-C: The Causal Failure and Lessons for Diagnostic Frameworks

The canonical observational inverse association between HDL-C and cardiovascular disease was tested by MR using variants specifically elevating HDL-C. Voight et al. demonstrated in 2012 that genetic HDL-C elevation did not reduce coronary heart disease risk, predicting, correctly, the clinical failure of CETP inhibitors designed to raise HDL-C. This finding, while directly relevant to neither FH nor FHTG diagnosis, established a methodological principle of considerable importance: observational associations, however robust, require MR validation before being incorporated into diagnostic and therapeutic frameworks. The belief that a biomarker may be a marker of metabolic health rather than a causal therapeutic target applies with equal force to the current use of LDL-C as the single diagnostic criterion for FH.

#### 2.5.6. Critical Appraisal of Current Diagnostic Criteria: Identified Gaps and Systematic Limitations

##### Age-Dependent Confounding of LDL-C in Adults

The most pervasive limitation of current FH diagnostic criteria is the age-dependent erosion of LDL-C diagnostic specificity. In the general non-FH population, LDL-C increases gradually with age due to declining LDL receptor activity, hormonal changes (particularly post-menopausal estrogen withdrawal in women), progressive weight gain, and cumulative dietary habits [54,55,56,57]. By age 30–35, these acquired changes can produce LDL-C concentrations ranging between 190 and 230 mg/dL in otherwise genetically normal individuals, precisely the range that generates substantial DLCN points.

This age-dependency produces two opposing diagnostic errors. Among younger adults (under 30 years), the diagnostic signal-to-noise ratio for FH is highest; a genetically determined LDL-C of 200 mg/dL in a 25-year-old has high specificity for monogenic FH. In older adults (over 40–45 years), the same absolute LDL-C level increasingly reflects polygenic susceptibility compounded by lifestyle factors. The DLCN does not include an age-correction algorithm for LDL-C points, nor does any other internationally endorsed scoring system [6,12].

Lp(a) does not share this limitation. Its plasma concentration is determined almost entirely by the LPA gene kringle IV type 2 repeat polymorphism, reaches adult levels by early childhood, and remains stable across the adult lifespan independent of diet, weight, or hormonal status [4,24]. This temporal and environmental stability makes Lp(a) a fundamentally more reliable genetic marker at any age above 5 years, yet it is absent from all current FH diagnostic criteria.

##### The Lp(a) Phenocopy Problem and Systematic Misclassification

The Lp(a) phenocopy represents one of the clinical diagnostic errors, in contemporary lipidology, leading to severe consequences. Because Lp(a) particles contain an LDL-like moiety, elevated Lp(a) contributes to the total LDL-C as measured by standard assays, typically adding approximately 0.3 mg/dL of LDL-C per 1 mg/dL of Lp(a) [6]. A patient with Lp(a) of 150 mg/dL, a level achievable by approximately the top 5% of the population, will have their apparent LDL-C inflated by approximately 45 mg/dL depending on their true LDL-C values.

The clinical consequence is that DLCN scores based on uncorrected LDL-C may classify a patient as Probable or Definite FH when the actual underlying condition is isolated elevated Lp(a)—a genetically distinct disorder with different pathophysiology, different cardiovascular risk profile (particularly for aortic valve stenosis and calcification), and different treatment targets (Lp(a) is not meaningfully lowered by statins) [7,51]. Studies using post hoc correction of LDL-C for Lp(a) content suggest that up to 25% of clinically diagnosed FH cases are attributable in whole or partially to elevated Lp(a) rather than LDLR pathway dysfunction [3].

##### Failure to Discriminate Monogenic from Polygenic Etiologies

The binary categorical structure of the DLCN and Simon Broome criteria cannot distinguish between monogenic FH, conferring a lifetime risk of coronary heart disease approaching 50% in men aged 50 without treatment, and polygenic hypercholesterolemia, in which cumulative LDL-C from multiple common variants produces a similar lipid phenotype, but a substantially lower absolute cardiovascular risk [22,41]. Trinder and colleagues demonstrated in 2020 that the presence of a monogenic FH mutation confers a risk of atherosclerotic cardiovascular disease that is 2.3-fold higher than that associated with matched polygenic hypercholesterolemia, even at equivalent LDL-C levels [22].

This is not a trivial distinction. Monogenic FH patients derive the greatest absolute benefit from aggressive, early, lifelong LDL lowering treatment, including PCSK9 inhibitor therapy. Polygenic phenocopies, while still at elevated risk, may be appropriately managed with less intensive regimens. The misclassification of polygenic phenocopies as monogenic FH inflates the apparent prevalence of FH while simultaneously directing maximum-intensity therapy toward patients who may not require it, with associated pharmacoeconomic and safety implications.

##### Inapplicability to Pediatric Populations

The DLCN was designed for and validated in adult populations. Its high-weight items (premature CHD events, corneal arcus before age 45, tendon xanthomas, and extreme LDL-C levels) are either absent or not age-normalized in pediatric patients. A child of a known FH patient who has inherited the mutation will typically present with LDL-C between 160 and 300 mg/dL but will score minimally on the DLCN, which cannot award points for events that have not yet occurred [32,58,59].

International guidelines increasingly recommend universal lipid screening between ages 9 and 11, before the pubertal hormonal shifts that complicate cholesterol measurement [3,4]. The DLCN, applied at this age, identifies only the most extreme phenotypes and systematically under-diagnoses the majority. The Simon Broome criteria offer somewhat better pediatric applicability due to their emphasis on family history, but remain incompletely validated for genetic endpoints in children.

##### Diagnostic Limitations Specific to FHTG

For FHTG, the diagnostic challenges are distinct but no less problematic. Unlike FH, FHTG lacks internationally endorsed diagnostic criteria of equivalent rigor. Clinical identification has relied predominantly on triglyceride thresholds (≥5.6 mmol/L or ≥500 mg/dL for severe hypertriglyceridemia and ≥1.7 mmol/L or ≥150 mg/dL for moderate forms) without accounting for particle composition, genetic substrate, or the presence of metabolic syndrome as a secondary contributor [5,60].

The predominant cardiovascular risk in FHTG is mediated by remnant cholesterol in VLDL and intermediate-density lipoprotein particles rather than by the triglyceride mass itself, yet no existing diagnostic framework systematically measures or determines remnant cholesterol or ApoB in FHTG patients as a primary criterion [52,61]. Furthermore, the clinical presentation of FHTG is heavily modulated by diet, alcohol consumption, insulin resistance, hypothyroidism, and pregnancy, secondary factors that can elevate triglycerides by 2- to 3-fold above baseline, producing compound hypertriglyceridemia that may not resolve without addressing both the genetic susceptibility and the environmental trigger [5,62].

The distinction between monogenic familial chylomicronemia syndrome, which requires lifelong, extreme dietary fat restriction and potentially volanesorsen or fitusiran therapy, and polygenic FHTG managed primarily through lifestyle, fibrates, omega-3 fatty acids, and treatment of underlying metabolic drivers, is very important. The correct diagnosis has profound treatment implications, but currently depends on genetic testing that is unavailable in most clinical settings [5,63] (Table 2).

To historically ground the transition from phenotypic clustering to precision genomic medicine, it is valuable to contrast the distinct diseases once loosely aggregated under the obsolete moniker of “Hereditary Hyperlipidemia”. Table 3 details the diverging genetic, clinical, and therapeutic profiles that mandate separate handling in modern clinical practice.

##### Sex-Specific Diagnostic Gaps: The Dynamic Hormonal Influence on Female Lipid Percentiles

A profound limitation of existing diagnostic frameworks for inherited dyslipidemias is their rigid, sex-neutral application of absolute lipid thresholds, which creates a substantial diagnostic gap for women. Traditional criteria fail to account for the dynamic shifts in circulating estrogen and other sex hormones, which modulate lipid metabolism across the distinct biological phases of a woman’s life: puberty, pregnancy, menopause, and post-menopause. Failing to utilize stage-specific LDL−C and Lp(a) percentiles introduces systemic under- or over-diagnosis of genetic lipid disorders.

- Puberty: During the transition into adolescence, sex-specific differences in lipid kinetics become pronounced. While young males often exhibit a transient dip in HDL−C and a steady rise in atherogenic particles, adolescent females display distinct variations heavily modulated by the onset of cyclic ovarian activity. Applying a blanket pediatric cut-off without accounting for pubertal staging can obscure the early signal of heterozygous FH.
- Pregnancy: Gestation represents a state of physiological hyperlipidemia driven by placental hormones and IR, required to support fetal development. During the second and third trimesters, maternal LDL−C can increase by up to 50% from baseline, occasionally unmasking hidden polygenic tendencies or mimicking a monogenic FH phenotype. Conversely, because standard clinical scoring tools rely heavily on absolute LDL−C thresholds, a pregnant woman with baseline borderline hypercholesterolemia might temporarily meet “Definite FH” criteria based solely on gestational spikes. Conversely, true FH patients may face diagnostic confusion if screening is inappropriately timed during pregnancy or immediately postpartum.
- Menopause and Post-Menopause: The transition through menopause marks the most diagnostically confounding era for women. The abrupt withdrawal of endogenous estrogen, which normally upregulates hepatic LDL re-expression, leads to a sharp increase in LDL−C concentrations, often by 10% to 20%. In women over 50, this age- and hormone-dependent surge erodes the specificity of tools like the DLCN score. Acquired, post-menopausal lipid elevations frequently generate high point totals, leading to the misclassification of lifestyle-compounded, age-related hypercholesterolemia as a primary genetic disorder.

##### Ancestry and Ethnicity Bias: The Misconception of Universal Diagnostic Thresholds

A major systemic limitation of contemporary clinical scoring tools is their reliance on absolute diagnostic thresholds validated almost exclusively in cohorts of European descent. Genetic dyslipidemias manifest against highly divergent, ancestry-specific baseline distributions of atherogenic and triglyceride-rich lipoproteins. Consequently, applying uncalibrated Eurocentric criteria universally results in severe diagnostic disparities, leading to both under-diagnosis and inappropriate risk stratification in non-European populations.

- Lipoprotein(a) Disparities: The genetic architecture of the LPA gene locus is highly sensitive to ancestry. Individuals of African descent exhibit median Lp(a) concentrations that are two- to threefold higher than those of European, Hispanic, or East Asian populations, with distinct shifts in the population percentiles. Conversely, East Asian populations display significantly lower median Lp(a) levels but face an equivalent or greater relative cardiovascular hazard per unit increase. Using a single, static threshold (e.g., >30 mg/dL or >50 mg/dL) fails to capture the true genetic risk across diverse populations, disproportionately misclassifying individuals of African descent as Lp(a) “phenocopies” or underestimating the pathology in East Asians.
- Triglycerides and ApoB Variation: Marked ethnic variations exist in the phenotypic expression of hypertriglyceridemia and its particle-centric correlates (ApoB and remnant cholesterol). For instance, South Asian populations characteristically exhibit a highly atherogenic dyslipidemia triad, marked by elevated triglycerides and high ApoB particle numbers at lower absolute LDL−C levels, driven by an interaction of polygenic susceptibility and IR. Conversely, individuals of African descent frequently display lower fasting triglyceride concentrations even in the presence of severe metabolic syndrome. Uncalibrated, triglyceride-centric definitions of FHTG systematically miss high-risk patients in populations where remnants are heavily enriched, but absolute triglyceride concentrations remain below standard clinical cut-offs.
- LDL-C and Atherogenic Particle Mismatch: The core component of classic FH criteria (unadjusted absolute LDL−C concentration) does not reflect an identical particle burden or coronary hazard across distinct ethnic cohorts. Because absolute LDL−C distributions are shifted by environmental, dietary, and genetic backgrounds, a threshold of 190 mg/dL carries different positive predictive values for a monogenic defect depending on the patient’s ancestral background, emphasizing the urgent clinical need for ethnicity-adjusted baseline percentiles.

##### The Special Case of Lp(a) Fluctuation in Women

While plasma Lp(a) concentrations are predominantly determined genetically by the LPA gene locus and remain highly stable throughout a man’s life, women present a critical exception. Lp(a) levels are highly sensitive to significant drops in estrogen. Clinical evidence demonstrates that Lp(a) percentiles frequently shift upward during pregnancy and, most notably, experience a significant, sustained increase (up to 10–15%) following natural or surgical menopause.

Because elevated Lp(a) contributes directly to measured total LDL−C (creating the “phenocopy problem” detailed in Section The Lp(a) Phenocopy Problem and Systematic Misclassification), the post-menopausal surge in both Lp(a) and LDL−C compounds the risk of diagnostic error. A woman who was phenotypically “invisible” or classified as “unlikely FH” in her 30s may suddenly cross the diagnostic threshold in her late 50s due entirely to hormonal restructuring, rather than a monogenic defect.

##### Mortality and Morbidity for FH, Respectively, FHTG

Cardiovascular mortality remained significantly elevated in FH patients, around 2.3 versus the general population for individuals younger than 70 years. All-cause mortality was not significantly increased compared with the general population. Cardiovascular disease accounted for approximately 46% of all deaths among FH patients. By the 2010s, event rates had fallen substantially compared with earlier decades because most diagnosed patients received long-term lipid-lowering treatment. Nevertheless, FH remained one of the strongest inherited risk factors for atherosclerotic cardiovascular disease [66,67].

Classic FHTG does not appear to be associated with markedly increased premature cardiovascular mortality when isolated from other lipid abnormalities. The principal complications remained acute pancreatitis, chylomicronemia syndrome, and severe hypertriglyceridemia-related symptoms [42,68,69].

##### Specific Therapies for FH and FHTG for These Phases

For FH, high-intensity statins (rosuvastatin and atorvastatin) were first-line therapy. If LDL-C target levels were not reached, ezetimibe was added. A major breakthrough in the therapy of FH represented the discovery of PCSK9 inhibitors (evolocumab and alirocumab). Approved in 2015, these monoclonal antibodies could reduce LDL cholesterol by an additional 50–60% beyond statin therapy and transformed the management of severe heterozygous FH. Other therapies for homozygous FH were lomitapide and mipomersen. It was approved in the United States in 2013 for homozygous FH, though its use remained limited because of adverse effects and liver toxicity concerns. LDL apheresis continued to be used, especially in homozygous FH or in severe heterozygous FH refractory to drug therapy [3,4,70].

For FHTG, the main therapies remained fibrates (fenofibrate and gemfibrozil) and omega-3 fatty acids, especially in severe cases. Statins were added particularly when hypertriglyceridemia coexisted with elevated LDL-C or increased cardiovascular risk [71,72].

#### 2.5.7. Future Directions: Toward an Integrated, Genomically Informed Diagnostic Framework

##### Mandatory Lifetime Lp(a) Measurement and LDL-C Correction

The most urgent update to current FH diagnostic criteria and practice is the universal incorporation of Lp(a) measurement. The European Society of Cardiology (ESC/EAS) guidelines already recommend at least one lifetime Lp(a) measurement for every adult [3,4,7]; however, this recommendation has not yet been integrated into FH diagnostic algorithms. The next update of DLCN or equivalent criteria should correct LDL-C for Lp(a) contribution using validated formulas (subtracting approximately 30% of Lp(a) mass in mg/dL from total LDL-C) before scoring, and should include an independent Lp(a) risk module that identifies patients with isolated Lp(a) elevation, neither diagnosing them as FH nor dismissing their elevated risk [6,7].

Beyond its diagnostic role, Lp(a) measurement will become therapeutically essential as RNA-interference agents targeting LPA (including pelacarsen, olpasiran, zerlasiran, and lepodisiran) are analyzed in Phase 3 clinical trials [7]. Patients with Lp(a)-mediated phenocopies of FH require Lp(a)-targeted, rather than LDL-targeted, therapy. Without a diagnostic framework that separates these populations, appropriate therapy cannot be initiated.

##### Polygenic Risk Scoring as a Complement to Phenotypic Criteria

The incorporation of validated polygenic scores (PGSs) into FH diagnostic algorithms represents the next major methodological advance [37,38,73,74]. A validated LDL-C polygenic score, applied at the point of diagnosis, can identify patients whose elevated LDL reflects many common small-effect variants rather than a monogenic defect, reclassifying them from Definite FH to polygenic hypercholesterolemia with significant differences in prognosis and treatment intensity [22,41].

Technically, genome-wide single-nucleotide polymorphism (SNP) arrays capable of generating a polygenic score are increasingly accessible and cost-comparable to conventional genetic mutation panels. The analytical challenge is standardization: polygenic scores must be validated across diverse ethnic populations, as most current PGSs were derived from European-ancestry cohorts and show attenuated performance in South Asian, African, and East Asian populations [75]. Future diagnostic frameworks must specify ancestry-appropriate PGS thresholds or develop trans-ethnic scores with demonstrated performance across various populations.

##### Resolving the Polygenic Risk Score Equity Gap

While the incorporation of PGS represents a major methodological leap forward, standardizing these tools across ancestrally diverse populations remains a primary challenge. Because most current LDL-C polygenic scores were derived from European-ancestry cohorts, they exhibit significantly attenuated diagnostic and predictive performance when applied to South Asian, African, and East Asian populations.

To prevent the exacerbation of global health disparities, future integrated diagnostic frameworks must reject a “one-size-fits-all” approach. Precision lipidology mandates the deployment of trans-ethnic polygenic risk scores and the establishment of ancestry-specific, percentile-based reference values for LDL−C, ApoB, Lp(a), and TG. Transitioning from absolute European-centric cut-offs to calibrated, population-specific percentiles is an essential prerequisite for equitable, personalized cardiovascular prevention.

##### Precision Lipidology Requires Stage-Specific Diagnostics

To bridge this gap, future diagnostic paradigms must transition from static phenotypic cut-offs to dynamic, sex-specific, and hormone-stage-adjusted lipid percentiles. Integrating reference ranges calibrated specifically to a woman’s hormonal status, paired with mandatory Lp(a) correction and front-line genomic confirmation, is essential to optimize the precision of diagnostic algorithms, preventing both the under-treatment of high-risk younger women and the inappropriate over-diagnosis of post-menopausal individuals. 

##### ApoB as the Unifying Diagnostic Biomarker

Given the MR evidence that ApoB is the primary causal determinant of atherosclerotic cardiovascular disease (ASCVD) across all atherogenic lipoprotein fractions, ApoB measurement should be incorporated as a mandatory element of both FH and FHTG diagnostic work-up [48,49]. For FH, ApoB provides a particle-level assessment of the LDL burden that is not distorted by Lp(a) and better reflects the true atherogenic exposure than LDL-C. For FHTG, ApoB captures the total remnant-enriched particle burden and is a more reliable predictor of residual cardiovascular risk than triglycerides or non-HDL cholesterol alone [48].

A future integrated diagnostic criterion might define FH as: (1) LDL-C above age-adjusted threshold after Lp(a) correction, or (2) ApoB above 130 mg/dL with confirmed family history or genetic mutation, in the absence of secondary causes. This dual-pathway criterion would increase sensitivity without sacrificing specificity and would capture the particle-level biology that LDL-C alone cannot.

##### Genomic Cascade Testing as the Diagnostic Gold Standard

For both FH and FHTG, the progressive reduction in sequencing costs is shifting the question from “Can we afford genetic testing?” to “Can we afford not to perform genetic testing?” For FH specifically, the identification of a causal LDLR, APOB, or PCSK9 mutation definitively confirms the diagnosis, removes phenotypic ambiguity, enables cascade screening of first- and second-degree relatives (who carry a 50% prior probability of the same mutation), and stratifies risk by mutation type—severe LDLR null mutations conferring higher risk than APOB mutations or gain-of-function PCSK9 variants [4,76,77].

For FHTG, sequencing of LPL, APOC2, APOA5, LMF1, and glycosylphosphatidylinositol-anchored high-density lipoprotein-binding protein 1 (GPIHBP1) enables identification of the minority with true monogenic FCS, who require fundamentally different management (volanesorsen, fitusiran) compared to polygenic FHTG patients. A future diagnostic framework for severe hypertriglyceridemia should mandate genetic sequencing for all patients with fasting triglycerides persistently exceeding 10 mmol/L, in whom monogenic FCS must be excluded before initiating therapy [8,42,78].

##### Redefining FHTG: From Triglyceride Thresholds to Particle-Centric Criteria

The redefinition of FHTG requires a conceptual shift from triglyceride concentration as the primary diagnostic variable to a composite criterion incorporating remnant cholesterol (or non-HDL cholesterol as a practical surrogate), ApoB, and genetic substrate. A future FHTG diagnostic framework should categorize patients along two independent axes: (1) severity of triglyceridemia, distinguishing moderate (2–10 mmol/L) from severe (≥10 mmol/L) hypertriglyceridemia; and (2) genetic architecture, distinguishing polygenic from oligogenic or monogenic FCS, with implications for both pancreatitis risk management and ASCVD risk stratification [5,52,79].

The therapeutic implications of this two-axis framework are substantial. Patients with severe monogenic FHTG require immediate triglyceride reduction to prevent pancreatitis, prioritizing agent choice over ASCVD benefit. Patients with moderate polygenic FHTG and elevated remnant cholesterol or ApoB require therapy directed primarily at ASCVD prevention, with triglyceride-lowering as a secondary objective. Current guidelines do not fully articulate this distinction in algorithmic form [4].

##### Integration of Early Pediatric Screening into Diagnostic Pathways

The biological rationale for pediatric FH screening is compelling: FH is present from birth, atherosclerosis begins accelerating in the second decade in untreated heterozygotes, and statin therapy initiated in childhood is safe, effective, and produces incremental benefits on the carotid intima–media thickness that otherwise worsens during adult life [80,81]. Universal screening between ages 9 and 11, at which point LDL-C is measurable but largely unconfounded by pubertal hormonal variation, should be paired with a pediatric-specific FH criterion that uses age- and sex-adjusted LDL-C thresholds and weighs family history more heavily than physical signs that have not yet manifested [58].

For FHTG, pediatric screening is less well-evidenced but should be considered in children of parents with severe hypertriglyceridemia, particularly where monogenic FCS is suspected, given the risk of dietary-trigger-induced pancreatitis in adolescence [5,82,83,84].

#### 2.5.8. Proposed Integrated Diagnostic Model for Future Application

**Figure 2:**
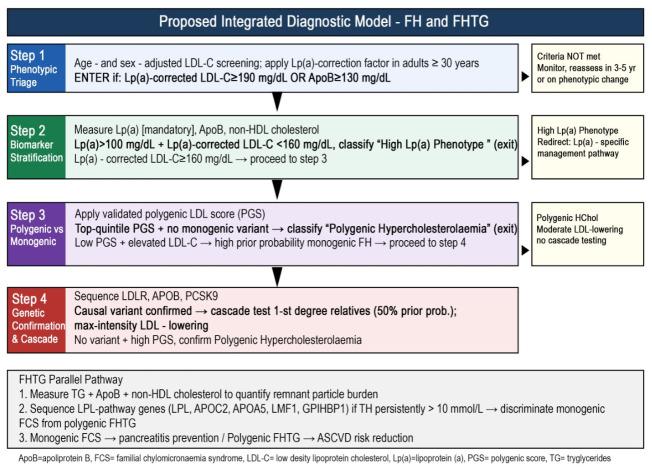
Proposed four-step integrated diagnostic algorithm for familial hypercholesterolemia (FH) and parallel pathway for familial hypertriglyceridemia (FHTG). Sequential steps progress from phenotypic triage (Step 1) through biomarker stratification (Step 2), polygenic versus monogenic discrimination (Step 3), to genetic confirmation and cascade screening (Step 4). Lateral exits denote alternative diagnostic classifications at each decision node. The FHTG pathway applies analogous logic using triglyceride burden, remnant cholesterol, ApoB, and LPL-pathway sequencing. ApoB = apolipoprotein B; FCS = familial chylomicronemia syndrome; LDL-C = low-density lipoprotein cholesterol; Lp(a) = lipoprotein(a); PGS = polygenic score; TG = triglycerides.

Drawing together the historical evolution, identified gaps, and emerging evidence reviewed above, an integrated diagnostic model for FH can be conceptualized around four sequential steps, applicable across the adult and pediatric population:

- Step 1—Phenotypic Triage. The algorithm is entered when age- and sex-adjusted LDL-C screening identifies a candidate. In adults aged 30 years or over, the 2022 European Atherosclerosis Society (EAS) consensus mandates application of an Lp(a) correction factor prior to diagnostic scoring, given the well-documented inflation of measured LDL-C by Lp(a) cholesterol content [7,33]. Patients with Lp(a)-corrected LDL-C ≥ 190 mg/dL (4.9 mmol/L) or ApoB ≥ 130 mg/dL proceed to Step 2; those below both thresholds are returned to standard surveillance with reassessment at 3–5 year intervals or upon phenotypic change (see Figure 2) [1,21].
- Step 2—Biomarker Stratification. Mandatory measurement of Lp(a), ApoB, and non-HDL cholesterol is performed. If Lp(a) exceeds 100 mg/dL (250 nmol/L)—a threshold associated with substantially elevated cardiovascular risk independent of LDL-C [7,24]—and the Lp(a)-corrected LDL-C falls below 160 mg/dL, the dominant atherogenic driver is Lp(a) rather than LDL receptor pathway dysfunction. These patients are classified as the “High Lp(a) Phenotype” and redirected to an Lp(a)-specific management algorithm [7]. Patients with corrected LDL-C ≥ 160 mg/dL and elevated ApoB consistent with excess atherogenic particle number [25] advance to Step 3 (see Figure 2).
- Step 3—Polygenic versus Monogenic Stratification. A validated weighted polygenic LDL score is applied. Talmud et al. demonstrated that a six-single-nucleotide-polymorphism LDL gene score accurately separates polygenic from monogenic FH in clinically ascertained populations [17], a finding replicated and extended by Khera et al. [18] and Trinder et al. [16]. Patients in the top quintile of polygenic score with no identifiable rare monogenic variant are classified as polygenic hypercholesterolemia and receive moderate treatment intensification without cascade testing. A low polygenic score in the context of persistent severe hypercholesterolemia implies high prior probability of a dominant monogenic variant affecting LDLR, APOB, or PCSK9, and prompts progression to Step 4 (see Figure 2) [31,47,48].
- Step 4—Genetic Confirmation and Cascade Screening. Sequencing of LDLR, APOB, and PCSK9 is performed. Identification of a pathogenic or likely pathogenic variant confirms monogenic FH and triggers cascade testing of first-degree relatives, each carrying a 50% prior probability of the variant [13,49]. Maximum-intensity LDL lowering therapy is initiated regardless of baseline LDL-C, consistent with evidence that lifetime exposure reduction is the dominant determinant of ASCVD benefit [19,20]. Patients without an identifiable monogenic variant but with high polygenic burden are reclassified as polygenic hypercholesterolemia; those with neither a monogenic variant nor an elevated polygenic score merit re-evaluation of secondary causes (see Figure 2) [11,18].

FHTG Parallel Pathway. For familial hypertriglyceridemia, the analogous framework requires triglyceride measurement paired with ApoB and non-HDL cholesterol to quantify remnant particle burden—the atherogenically relevant variable identified in Mendelian randomization analyses [23,24]. Targeted LPL-pathway gene sequencing (LPL, APOC2, APOA5, LMF1, GPIHBP1) is applied when triglycerides persist above 10 mmol/L, discriminating monogenic familial chylomicronemia syndrome from polygenic FHTG [25,32]. This distinction carries direct therapeutic significance: pancreatitis prevention is the primary objective in FCS, whereas ASCVD risk reduction governs management of polygenic FHTG [8,25].

This model is aspirational in its current technical and resource requirements; implementation at the primary care scale requires point-of-care polygenic scoring, affordable next-generation sequencing, and electronic health record integration of multi-variable diagnostic algorithms. Nevertheless, the evidence base reviewed here strongly supports its scientific validity [16,17,18,30], and the progressive cost reduction in genomic technologies makes implementation a realistic near-term prospect rather than a distant theoretical advance (see Figure 2).

For FHTG, the analogous model requires triglyceride measurement paired with ApoB and non-HDL cholesterol to assess remnant particle burden; targeted genetic sequencing of LPL-pathway genes for triglycerides persistently ≥ 10 mmol/L; and stratification into monogenic FCS versus polygenic FHTG pathways.

This model is aspirational in its current technical and resource requirements. Implementation in primary care settings at scale requires point-of-care polygenic scoring, affordable sequencing, and electronic health record integration of multi-variable diagnostic algorithms. However, the evidence base reviewed here strongly supports its scientific validity, and the progressive cost reduction in genomic technologies makes implementation a realistic near-term prospect rather than a distant theoretical advance.

## 3. Conclusions

Although HF and HFTG are not the only pathologies included in “Hereditary Hyperlipidemia”, they are the best-known and the most researched of these diseases. Thus, FH and FHTG are rare, autosomal-dominant inherited disorders; they are the most frequently encountered in clinical medicine. FH is characterized by very high LDL-C levels and normal or mildly elevated TG. Untreated FH is associated with an increased incidence of premature ASCVD, leading to cardiovascular death. FHTG is characterized by extremely elevated triglyceride values, normal or mildly increased LDL-C. Although the mortality for patients with untreated FHTG is less compared to FH, the morbidity is very high.

The diagnostic definition of FH has evolved over seven decades from a simplistic phenotypic clinical impression to a mechanistically grounded, genotype-informed construct. Yet the core scoring tools in global clinical use (the DLCN, Simon Broome, and MED-PED criteria) remain anchored to the phenotypic era, capturing the cholesterol-centric surrogate of a disorder that is fundamentally genomic and particle-centered.

The evidence reviewed here converges on a clear conclusion: LDL-C, while an adequate triage variable, is insufficient as the primary diagnostic criterion for FH in adult populations. It is confounded by age, lifestyle, and Lp(a) values. It cannot discriminate monogenic from polygenic etiologies, and fails to capture the particle-level biology that MR studies identify as the primary causal driver of cardiovascular risk. Lp(a) measurement, ApoB quantification, polygenic risk scoring, and cascade genetic testing each address specific dimensions of this diagnostic gap and should be incorporated systematically into future criteria.

For FHTG, the redefinition is equally urgent. The shift from triglyceride concentration to remnant cholesterol and ApoB as the primary atherogenic variables, the genetic distinction between monogenic FCS and polygenic FHTG, and the therapeutic divergence between pancreatitis prevention and ASCVD reduction all require a diagnostic framework that current practice lacks.

The next decade will likely witness the convergence of affordable genomic sequencing, validated polygenic scores, and RNA-interference therapeutics for both LDL-C and Lp(a) in a way that makes diagnoses based on phenotype alone increasingly inadequate. The diagnostic frameworks of the 1990s served their era with distinction. The evidence now exists to build their successors.

## Figures and Tables

**Table 1:** Comparative overview of international FH diagnostic criteria. DLCN: Dutch Lipid Clinic Network; MED-PED: Make Early Diagnoses to Prevent Early Deaths [32,34].

Criterion	DLCN (The Netherlands)	Simon Broome (UK)	MED-PED (USA)
Scoring method	Numerical (weighted points)	Categorical (Definite/Possible)	LDL-C cut-offs by age/relationship
Physical signs required	Yes (high-weight items)	Yes (required for Definite)	No
Family history	Weighted points	Required for Possible FH	Implicitly (probands only)
Pediatric application	Limited	Limited	Age-adjusted (9–11 years)
Sensitivity for genotype	43–54%	~70% (estimates vary)	Lower (LDL-centric)
Lp(a) adjustment	None	None	None
Best clinical context	Adult population	UK primary/secondary care	Global population screening

Legend: FH—family hypercholesterolemia; Lp(a)—lipoprotein a; LDL—low-density lipoprotein; LDL-C—low-density lipoprotein cholesterol; UK—United Kingdom; USA—United States of America.

**Table 2:** Summary of identified diagnostic gaps and their evidentiary basis.

Limitation	Impact on FH Diagnosis	Impact on FHTG Diagnosis	Evidence Quality
Age-dependent LDL-C confounding	High (over-diagnosis in older adults)	Moderate (secondary TG elevation)	High (MR, longitudinal data)
Lp(a) phenocopy misclassification)	High (up to 25% misclassified)	Low (indirect)	High (MR and genetic studies)
Inability to distinguish mono vs. polygenic	High (different prognosis/treatment)	High (FCS vs. polygenic FHTG)	High [5,22]
Pediatric inapplicability	High (early screening failure)	Moderate	Moderate (consensus-based)
Absence of ApoB criterion	High (particle burden unmeasured)	High (remnant cholesterol)	Absence of ApoB criterion High (particle burden)
No remnant cholesterol criterion	Low	High (main ASCVD driver)	High [52]
No genetic cascade integration	High (relatives unscreened)	Moderate	High (EAS consensus)

Legend: ASCVD—atherosclerotic cardiovascular disease; EAS—European Atherosclerosis Society; FCS—familial chylomicronemia syndrome; TG—triglycerides; MR—Mendelian randomization.

**Table 3:** Characteristics of pathological entities historically clustered under “Hereditary Hyperlipidemia” [34,64,65].

Feature/Dimension	Monogenic Familial Hypercholesterolemia (FH)	Polygenic Hypercholesterolemia	Monogenic Familial Chylomicronemia Syndrome (FCS)	Polygenic Familial Hypertriglyceridemia (FHTG)
Primary Genetic Etiology	Autosomal-dominant loss-of-function in *LDLR* or *APOB*; gain-of-function in *PCSK9*.	Cumulative burden of common, small-effect LDL-raising single-nucleotide polymorphisms (SNPs).	Autosomal recessive homozygous/compound heterozygous mutations in *LPL*, *APOC2*, *APOA5*, *LMF1*, or *GPIHBP1*.	Polygenic susceptibility combined with metabolic stressors (obesity, diabetes, alcohol).
Pathophysiological Core	Impaired clearance of LDL particles from circulation, driving lifelong exposure from birth.	Modest reductions in hepatic clearance or overproduction of LDL, heavily influenced by age and lifestyle.	Near-complete absence of lipoprotein lipase activity; failure to clear dietary chylomicrons.	Hepatic overproduction of VLDL and/or partial impairment of triglyceride-rich lipoprotein catabolism.
Primary Clinical Stigmata	Tendon xanthomas, corneal arcus before age 45, and rapidly accelerated premature coronary heart disease.	Absence of classical physical stigmata; cardiovascular risk matches population curves or mild acceleration.	Eruptive xanthomas, lipemia retinalis, recurrent acute pancreatitis, and hepatosplenomegaly.	Variable; can present as metabolic syndrome, eruptive xanthomas (if TG extreme), and increased coronary disease risk.
Contemporary Diagnostic Criteria	DLCN score, Simon Broome “Definite”, or positive target next-generation sequencing.	Elevated absolute LDL-C, high polygenic risk score (top quintile), and negative monogenic testing panel.	Fasting triglycerides persistently > 10 mmol/L or >880 mg/dL, low/absent LPL activity, and monogenic verification.	Triglyceride thresholds > 1.7 mmol/L or >150 mg/dL, elevated ApoB, and prominent polygenic score component.
Historical Treatment Paradigms (Pre-1990s)	Extreme dietary fat/cholesterol restriction, bile acid sequestrants (Cholestyramine), partial ileal bypass surgery, and early plasma apheresis.	Standard low-fat dietary modifications and empirical nicotinic acid or fibrate regimens.	Drastic, life-altering restriction of dietary fat (<15–20 g/day).	Caloric restriction, alcohol cessation, and early first-generation fibrates or niacin.
Modern and Emerging Therapeutic Targets	High-intensity statins, ezetimibe, PCSK9 inhibitors, and upcoming anti-sense oligonucleotides/RNA (e.g., mipomersen).	Statins, ezetimibe, or standard oral lipid-lowering therapies adjusted to absolute baseline cardiovascular risk calculations.	RNA-interference and antisense technologies targeting inhibitors of alternative pathways (e.g., volanesorsen).	Lifestyle optimization, highly selective PPAR agonists (fibrates), high-dose prescription omega-3 fatty acids, and treating insulin resistance.

Legend: APOA5—gene expressing apolipoprotein A5, APOC2—gene expressing apolipoprotein C2; DLCN—Dutch Lipid Clinic Network; GPIHBP1—glycosylphosphatidylinositol-anchored high-density lipoprotein-binding protein 1; PCSK9—proprotein convertase subtilisin/kexin type 9; LDL—low-density lipoproteins; LMF1—lipase maturation factor 1; LPL—lipoprotein lipase; RNA—ribonucleic acid; PPAR—peroxisome proliferator-activated receptors; SNPs—single-nucleotide polymorphisms; VLDL—very low-density lipoprotein.

## Data Availability

All data are included in the text.

## Abbreviations

The following abbreviations are used in this manuscript:

APOB	The gene coding ApoB
ASCV	Atherosclerotic Cardiovascular Disease
DLCN	Dutch Lipid Clinic Network
FH	Familial Hypercholesterolemia
FHTG	Familial Hypertriglyceridemia
HDL	High-Density lipoprotein
Lp(a)	Lipoprotein (a)
LDL	Low-Density Lipoprotein
MR	Mendelian Randomization
MVMR	Multi-variable MR
PCSK9	Proprotein Convertase Subtilisin/Kexin type 9
PGS	Polygenic Score
VLDL	Very Low-Density Lipoprotein
